# Low-Cost GRIN-Lens-Based Nephelometric Turbidity Sensing in the Range of 0.1–1000 NTU

**DOI:** 10.3390/s18041115

**Published:** 2018-04-06

**Authors:** Michael Metzger, Alexander Konrad, Felix Blendinger, Andreas Modler, Alfred J. Meixner, Volker Bucher, Marc Brecht

**Affiliations:** 1 Institute for Applied Research, Faculty for Mechanical and Medical Engineering, Furtwangen University, 78054 Villingen-Schwenningen, Germany; michael.metzger@hs-furtwangen.de (M.M.); felix.blendinger@hs-furtwangen.de (F.B.); volker.bucher@hs-furtwangen.de (V.B.); 2 Institute of Physical and Theoretical Chemistry, University of Tuebingen, 72076 Tuebingen, Germany; alexander.konrad@uni-tuebingen.de (A.K.); alfred.meixner@uni-tuebingen.de (A.J.M.); 3 Department of Mathematics, Physics and Chemistry, Beuth University of Applied Sciences, 13353 Berlin, Germany; Andreas.Modler@beuth-hochschule.de; 4 Process Analysis and Technology (PA&T), Reutlingen Research Institute, Reutlingen University, 72762 Reutlingen, Germany

**Keywords:** turbidity sensing, nephelometric turbidimeter, nephelometer, formazin, water monitoring

## Abstract

Turbidity sensing is very common in the control of drinking water. Furthermore, turbidity measurements are applied in the chemical (e.g., process monitoring), pharmaceutical (e.g., drug discovery), and food industries (e.g., the filtration of wine and beer). The most common measurement technique is nephelometric turbidimetry. A nephelometer is a device for measuring the amount of scattered light of suspended particles in a liquid by using a light source and a light detector orientated in 90° to each other. Commercially available nephelometers cost usually—depending on the measurable range, reliability, and precision—thousands of euros. In contrast, our new developed GRIN-lens-based nephelometer, called GRINephy, combines low costs with excellent reproducibility and precision, even at very low turbidity levels, which is achieved by its ability to rotate the sample. Thereby, many cuvette positions can be measured, which results in a more precise average value for the turbidity calculated by an algorithm, which also eliminates errors caused by scratches and contaminations on the cuvettes. With our compact and cheap Arduino-based sensor, we are able to measure in the range of 0.1–1000 NTU and confirm the ISO 7027-1:2016 for low turbidity values.

## 1. Introduction

Turbidity is the reduction of transparency due to the amount of scattered light that results from the interaction between a beam of light and matter. The intensity of scattered light from a single particle depends on the size and shape of the particle as well as on the wavelength of the incident light and the scattering angle, which can be described by a scattering coefficient [1]. If a mono-disperse particle solution is analyzed, the scattering intensity increases linearly over a wide range of (low) particle concentrations. As a consequence, the amount of scattered light can be used to determine the particle concentration. This fails with suspensions consisting of several particles with different sizes and shapes.

Turbidity can be analyzed by using turbidimeters, which normally consist of a collimated light source (mainly a laser or a light emitting diode (LED)), a sample chamber, and one or more detectors with a specific orientation to the illumination source [1,2]. The detected light intensity of every turbidimeter has to be converted to a turbidity unit, such as a Formazin Nephelometric Unit (FNU), a Formazin Turbidity Unit (FTU), or a Nephelometric Turbidity Unit (NTU), which depends on the sensor principle or setup, application field, and calibration [3]. Therefore, a calibration with different primary standards of known turbidity values must be performed. The basic setups of turbidimeters can be categorized by the type of the detection angle for the scattered light and the number of light detectors. Basically, three different turbidity sensing methods are used [1,2,3,4]: (i) a method where the transmitted intensity is analyzed with a turbidimeter with respect to the incident intensity, used for turbid samples with considerable turbidity values because a significant reduction of the incident light intensity is needed for precise results, (ii) a method by which a nephelometer measures the intensity of scattered light at 90°, which scales with the particle concentration in the sample, very common and very sensitive to a broad range of different particle sizes, the main advantage of which is the high accuracy at low turbidity levels, and (iii) the so-called ratio method, a combination of analyzing the transmitted light and the 90° scattered light with, normally, one or two more forward-scattered and back-scattered (e.g., 60° and 120°) signals, which is more precise than the others and allows for the measurement of a large range of turbidity values.

To analyze turbidity values in the most common range of 0.1–1000 NTU (e.g., for water analysis [1,5,6,7], process/analytical monitoring [8,9], and drug discovery [10,11]), the nephelometric concept is the best compromise due to the good price–performance ratio and its superlative cost-effectiveness. It combines a simple turbidity sensing setup with high precision, especially at lower turbidity levels. As a consequence, nephelometric turbidity measurements are one of the most common measurements used in the qualitative assessment of water suitability [12]. For drinking water, the turbidity value must not exceed 1.0 NTU ([13]), because the particles that cause turbidity are principal indicators for disinfection processes (distilled water: 0.08 NTU; tap water: 0.54 NTU; raw water: 3.52 NTU; water in rivers: up to 150 NTU; wastewater: about 1000 NTU [1,14,15]). Therefore, turbidity data is useful in drinking water treatment and production as well as wastewater and environmental monitoring [16,17,18].

Furthermore, nephelometric measurements are used in the chemical, pharmaceutical (filtration equipment monitoring [8,9,10,11]), and food industries (the filtration of wine and beer). For quality control or to distinguish between cloudy and clear juices, the turbidity measured with a nephelometer is also a key parameter [19,20,21].

Due to many possible applications, a considerable amount of peer-reviewed literature has emerged during the last few years describing new nephelometer designs and measurement setups. Sampedro et al. developed a new nephelometric turbidimeter in combination with an RGB sensor for obtaining additional information about the color of a sample [22]. Another low-cost nephelometer presented by Omar et al. uses also an IR-LED as a light source and detects within a range of 0–500 NTU [6]. Lambrou et al. presented a nephelometric turbidimeter using a red-laser for illumination, and a photodiode for detection measuring NTU values from 0 to 100 [2]. They also developed a low-cost system for measuring considerably high water quality parameters, including turbidity, temperature, pH, and conductivity [7]. Another new nephelometer applies an IR-LED and a light-to-frequency converter in combination with a microcontroller to detect the scattered light from 0 to 1000 NTU [18]. Tai et al. presented a further nephelometric sensor in the range from 0 to 100 NTU, using an IR-LED and a silicon photoelectric generator, and the sensor was calibrated with a self-synthesized formazin solution [23]. A further publication presented a combination of a temperature and turbidity measurement system for measurements of samples in an NTU range of 1–200 [24]. Hussain et al. developed a turbidimeter especially for analyzing water samples [25].

In this study, we present a new nephelometric turbidimeter, called GRINephy, for measuring within an NTU range of 0.1–1000 NTU, which conforms to the standard water analysis norm for low turbidity values (<400 NTU) [26]. The norm requires, for the NTU range of 0–400 NTU, a detection angle of 90° for the scattered light (nephelometric measurement) with respect to the incident beam, an aperture angle of a maximum of 30°, and an illuminating light ray at 860 nm with a limited convergency of 1.5°. For that reason, we used for illumination a fiber-coupled 860 nm SMD-LED, which was fused to the flat surface of a GRIN lens to emit collimated light. GRIN lenses achieve their focusing properties due to a continuous change of the refractive index within the lens material [27]. Therefore, the focus is on the plane surfaces of the lens, which allows for the possibility of glueing the optical fiber directly onto the lens, which is one of the main applications of GRIN lenses. Another GRIN lens collects scattered light and focuses it on a light-to-frequency sensor at a detection angle of 90°. GRIN lenses are also an alternative for standard objectives of obtaining optical properties with low costs. For detection, the aperture angle or the numerical aperture of the GRIN lens is reduced to 30° to conform to ISO 7027-1:2016 by adding an aperture between the GRIN lens and the sensor. According to this regulation, we calibrate and verify our GRINephy with a primary calibration standard called StablCal^®^ (stabilized formazin) and compare the results to a commercially available nephelometer (2100Qis, Hach, Loveland, CO, USA). Our GRINephy provides results in the range of 0.1–1000 NTU that are comparable to the commercial nephelometer. With this compact and cheap GRINephy, we are able to measure with excellent reproducibility and precision at very low turbidity levels by rotating the sample. Hence, we obtain average turbidity values determined by about 50 measured positions of the cuvettes.

## 2. Materials and Methods

**Figure 1:**
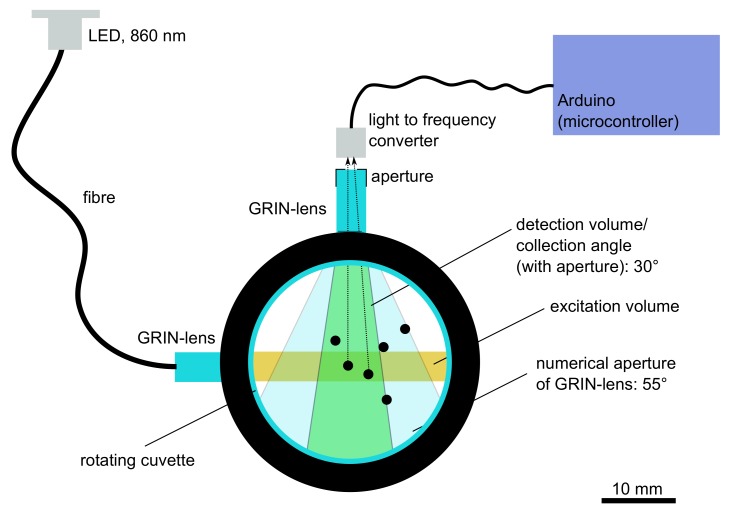
Sketch of GRINephy, which shows the main parts of the sensor including the illumination part, the detection unit, and the chamber parts.

Our nephelometric sensor system was built using a cylindrical self-designed polyoxymethylene (POM) cuvette holder that uses 25-mm-round glass cuvettes (2434706, Hach) (see Figure 1). For illuminating the sample, an SMD LED emitting at 860 nm (SFH 4253, Osram, Munich, Germany)in the near infrared region, which observes the European turbidity norm ISO 7027-1:2016, was used. The LED is coupled into a 0.2-mm-diameter-core fiber (FP200URT, Thorlabs, Newton, NJ, USA)and decoupled by a 0.25 pitch GRIN lens (W40–S0250–063–SBC, GoFoton, Somerset, NJ, USA)housed in the cuvette holder to guarantee parallel light with a 4 mm diameter. A second GRIN lens, at 90° with respect to the decoupling lens, collects the scattered light and focuses it toward a light-to-frequency sensor (TSL237, ams, Premstaetten, Austria). To reduce the detection angle, according to the European turbidity norm, to 20°–30°, a 2 mm aperture between the GRIN lens (normally 55°) and the detector was implemented. An 8-bit, 16 MHz microprocessor (Arduino Uno, Arduino, Turin, Italy) sums pulses from the sensor, which triggers an electrical pulse train with its frequency corresponding to the intensity of the detected light. This is done in different time intervals depending on the light intensity (normally 1 s). The same microprocessor controls a stepper motor (QSH4218-35-10-027, Trinamic, Hamburg, Germany) below the cuvette holder, which enabled us to rotate the sample. Thus, the position of the cuvette is turned automatically to 50 positions (always 7.2° further). At each position, the number of pulses are measured and saved in internal storage. A self-developed C-based Arduino program recognizes statistical outliers using a Grubbs’ test [28,29]. If no outliers are detected, the average of the 50 measured data values is determined and displayed. If one or more outliers are recognized, the measurement is considered as faulty and restarted from a different position (a maximum of 5 times). After starting at 5 different positions, further outliers (a maximum of 5) are considered, resulting in an average of at least 45 values. As a final result, we get a mean frequency value with a corresponding standard deviation. Otherwise, an error will be displayed and supposed existing bubbles should be removed or the cuvette should be changed. For calibration, we used the primary standard formazin with NTU values 0.1, 20, 200, and 1000 (StablCal^®^, Hach) and compared the results with a commercially available nephelometer (2100Qis, Hach) for validation. For this comparison, further NTU standards, with NTU values of 1, 10, 100, and 800 and some dilutions of 4000 NTU, were used.

## 3. Results

### 3.1. Calibration of GRINephy

**Figure 2:**
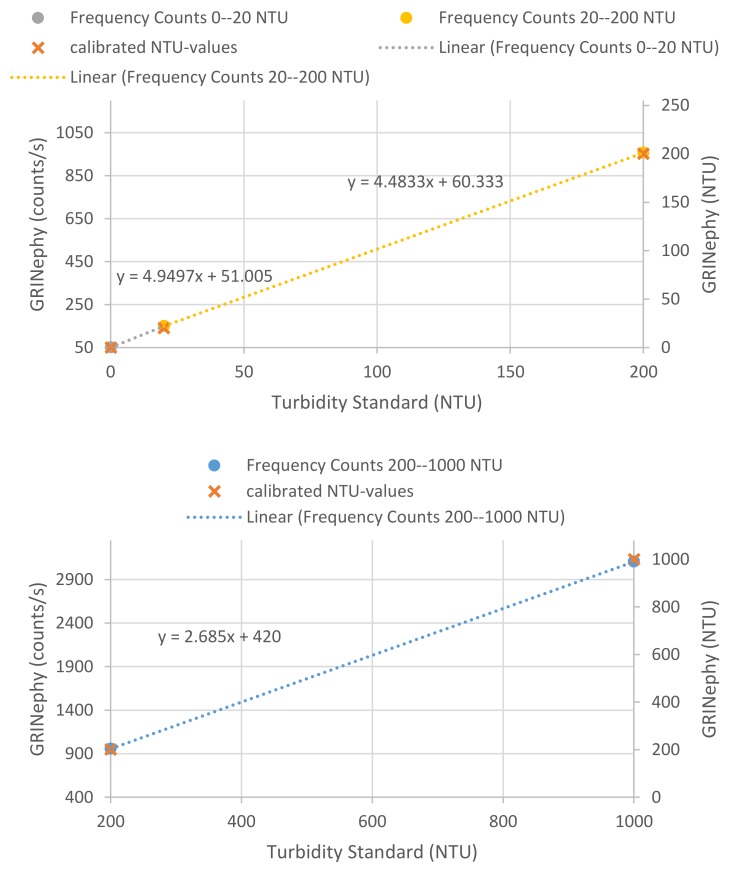
Calibration of GRINephy: (**top**) Output frequency signals of our GRINephy given in counts/s for measuring formazin turbidity calibration standards with the NTU values 0.1, 20, and 200 (gray and yellow dots). (**bottom**) Sub-region of 200–1000 NTU with the output frequency of our GRINephy using 200 and 1000 NTU calibration standards. The orange crosses show in both figures the converted NTU values.

The calibration of our GRINephy was performed by analyzing different calibration standards (StablCal^®^ Turbidity Standards Calibration Kit, 2662105, 0.1, 20, 200, and 1000 NTU, but not 4000 NTU, Hach). Therefore, each calibration solution was automatically measured on at least 45 positions on the cuvette by rotating the sample. As a final result, the mean values of the frequencies given by the light-to-frequency detector were determined, which is shown in Table 1 (middle column) and Figure 2. These frequency values were converted into the standard NTU values (0.1, 20, 200, and 1000 NTU). The total range of 0.1–1000 NTU was divided into the three sub-regions 0.1–20 NTU, 20–200 NTU, and 200–1000 NTU. For each sub-region, a linear regression was performed (see Figure 2). The *x*-axis values in Figure 2 correspond to the respective standard NTU values, and the *y*-axis represents the frequency values (gray, yellow, and blue dots) of the light-to-frequency detector and the converted NTU values (orange crosses).

### 3.2. Validation of GRINephy

**Figure 3:**
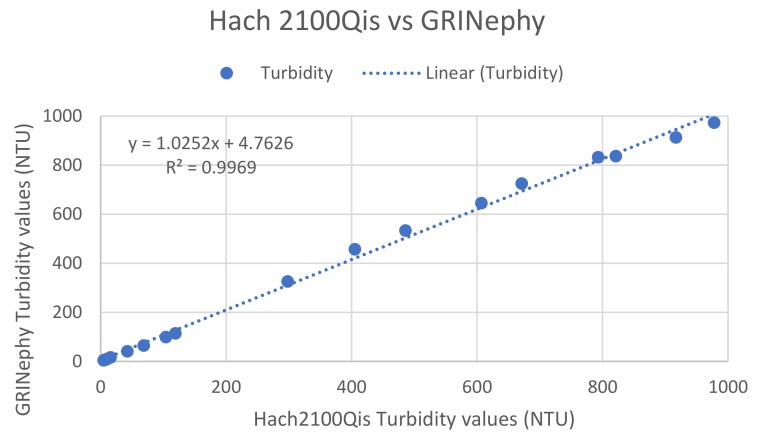
Validation of GRINephy: Values from the Hach2100Qis (*x*-axis) and the GRINephy (*y*-axis) using standard and diluted StablCal^®^ solutions.

The performance and operational capability of GRINephy was examined by comparing turbidity values measured and determined with our GRINephy with a commercially available nephelometer (Hach 2100Qis). Therefore, four solutions observing StablCal^®^ turbidity standards with NTU values of 1, 10, 100, and 800, and dilutions of the 4000 NTU standard calibration solution, were analyzed. The frequency values measured with GRINephy were converted into NTU values using the linear regression of the four calibration standards (see Figure 2). Table 2 summarizes the results for the measured solutions given by Hach2100Qis and our GRINephy. In Figure 3, one can see the determined turbidity values as a function of the turbidity values given by the Hach2100Qis with a Pearson’s r correlation coefficient of 0.9969.

## 4. Discussion

To our knowledge, this paper is the first to report a low-cost nephelometer with excellent reproducibility and precision for small turbidity values (<400 NTU) conforming to the ISO 7027-1:2016 norm. The illumination of the sample was done by an LED (860 nm) in combination with a GRIN lens instead of a laser due to the lower price, with lower energy consumption, and without a need to comply with laser safety regulations. The application of GRIN lenses guarantees parallel light for illumination and a limitation of the detection angle in collecting scattered light. This scattered light was detected by a light-to-frequency sensor, which is a cheap and simple method with high accuracy and linear-intensity-dependent behavior. The implementation of a stepper motor enables measurements at many different places to reduce disturbing parameters such as scratches and fingerprints on the cuvette by a special algorithm. Therefore, the reproducibility and precision, especially for low turbidity values (<5 NTU), was improved. Due to the fact that no regulation on a nephelometric turbidimeter calibration routine exists, the calibration of GRINephy was achieved with primary standard StablCal^®^ formazin solutions (with NTU values 0.1, 20, 200, and 1000 NTU). The choice of using three regression regions to characterize the calibration dataset presented in Figure 2 was motivated by a slight non-linearity in the dataset. To account for errors produced by the optical design, the measured values are divided into three sub-regions, which individually show an absolute linear behavior. Similar to Kelley et al., a buckling of linearity (at 200 NTU) was observed. The measured and analyzed data set of the GRINephy shows comparable results with respect to the commercially available device (see Table 2 and Figure 3). A plot of each mean value of all determined NTU values of the GRINephy against the mean NTU values recorded with the commercially available nephelometer shows an absolutely linear correlation, which indicates the applicability of GRINephy. The comparison of the standard deviation for each NTU value shows that the commercial device is slightly more precise and has a lower deviation (see Table 2). We developed a nephelometric sensor that measures in the range of 0.1–1000 NTU, as does the commercially available nephelometer. By measuring exclusively at 90°, we diverge from the norm for higher NTU values. Implementing a sensor at 0° the sensor to be further improved such that higher turbidity values conforming the norm can be achieved. Other low-cost nephelometers diverge partly from the norm and have further disadvantages, such as restricted accuracy for certain NTU ranges and the usage of a laser instead of an LED. Kelley et al. calibrated their device with a cutting oil solution instead of an accepted primary calibration standard such as formazin. The comparison between their nephelometer and the commercial one shows that there is a deviation in small turbidity values and no linearity in the range from 0 to 1000 NTU. A further nephelometric turbidity sensor presented by Sampredo et al. used only four self-made formazin solutions for calibration in the range of 0–500 NTU, and these solutions were not verified by independent measurement. Lambrou et al. used a laser for illumination and observed a deviation in their linearity. With this sensor, only values between 0 and 100 NTU could be analyzed. The sensor presented by Tai et al. was also only accurate within a range of 0–100 NTU. Hussein et al. claim that their developed sensor confirms to the ISO 7027-1:2016 norm, but they have no focusing element between the sample area and the detector. Therefore, the norm ISO 7027-1:2016 that stipulates that light be collected with angles less than 30° could not be fullfilled. In summary, the compact and cheap nephelometer presented here is optimal for measuring NTU values in the range of 0.1–1000 NTU with high repeatability and precision. This sensor has considerable scope and can be used by environmental organizations, public authorities, or private companies, or even by unaffiliated individuals. With small improvements and changes in the design, it is possible to take inline measurements, which would dramatically increase the scope of such types of sensors. In many turbidity sensing applications, continuous monitoring of the turbidity value by implementation of an inline system would simplify the process substantially.

## Figures and Tables

**Table 1:** Mean and standard deviation of measured StablCal^®^ standards with our GRINephy in counts per second (middle column) and the converted NTU values (right column).

StablCal^®^ Standard (NTU)	GRINephy (counts/s)	GRINephy (NTU)
0.1	51.5 ± 0.1	0.10 ± 0.01
20	150 ± 1	20.00 ± 0.2
200	957 ± 3	200 ± 1
1000	3105 ± 8	1000 ± 3

**Table 2:** Mean turbidity and standard deviation of measured standard StablCal^®^ solutions and dilutions with the commercial Hach2100Qis nephelometer and our GRINephy.

Sample	HACH2100Qis (NTU)	GRINephy (NTU)
Standard 1 NTU	1.09 ± 0.01	0.993 ± 0.04
dilution 1	4.50 ± 0.02	3.94 ± 0.08
dilution 2	7.91 ± 0.02	7.57 ± 0.09
Standard 10 NTU	10.3 ± 0.1	9.64 ± 0.1
dilution 3	15.8 ± 0.1	15.4 ± 0.1
dilution 4	42.6 ± 0.2	40.7 ± 0.2
dilution 5	68.6 ± 0.3	64.4 ± 0.3
Standard 100 NTU	104 ± 1	98.7 ± 0.5
dilution 6	119 ± 1	114 ± 1
dilution 7	298 ± 1	325 ± 1
dilution 8	405 ± 1	456 ± 1
dilution 9	486 ± 1	532 ± 1
dilution 10	607 ± 2	644 ± 2
dilution 11	671 ± 2	724 ± 2
dilution 12	793 ± 2	831 ± 2
Standard 800 NTU	821 ± 2	836 ± 2
dilution 13	917 ± 2	912 ± 3
dilution 14	978 ± 2	972 ± 3

## Abbreviations

The following abbreviations are used in this manuscript:

NTU	Nephelometric Turbidity Unit
ISO	International Organization for Standardization
FNU	Formazin Nephelometric Unit
FTU	Formazin Turbidity Unit
GRIN	gradient index
